# Effect of Sb Content on the Microstructure and Mechanical Properties of Eutectic SnPb Solder

**DOI:** 10.3390/ma17102233

**Published:** 2024-05-09

**Authors:** Xiuchen Zhao, Jiahui Chang, Xuefeng Wu, Zi-Ting Ye, Weiwei Chen, Xiaochen Xie

**Affiliations:** 1 School of Materials Science and Engineering, Beijing Institute of Technology, Beijing 100081, China; 3120211097@bit.edu.cn (J.C.); wuxuefeng0107@163.com (X.W.); ziting_ye@163.com (Z.-T.Y.); wwchen@bit.edu.cn (W.C.); 2 School of Integrated Circuit Science and Engineering, Beihang University, Beijing 100191, China; 3 Package R&D Center, Beijing Microelectronics Technology Institute, Beijing 100076, China

**Keywords:** eutectic SnPb solder, Sb addition, mechanical properties, creep resistance

## Abstract

SnPb solder was widely used in electronic packaging for aerospace devices due to its high reliability. However, its creep resistance is poor and can be improved by adding alloying elements. The effects of Sb content on the microstructure, tensile, and creep properties of eutectic SnPb solder were investigated. Sb addition effectively improved the mechanical properties of the SnPb solder. When Sb content exceeds 1.7 wt.%, SbSn intermetallic compounds (IMCs) occurred. And increasing the Sb content increased the tensile strength. Furthermore, Sb addition decreased the steady-state creep rate and increased the stress exponent *n*, suggesting that the creep resistance had been enhanced, which may be attributed to the hindrance of dislocation movement by SbSn IMCs, as well as the reduction in phase boundaries, which consequently reduced grain boundary sliding.

## 1. Introduction

SnPb solder was widely used in electronic devices due to its low melting temperature, good solderability, and low cost [1]. However, the Pb element is toxic, and the Restriction of Hazardous Substances (ROHS) banned the use of Pb in commercial electronic devices in 2006 [2]. Nevertheless, Pb-free solders may grow tin whiskers, leading to short-circuiting, which cannot meet the reliability requirements of aerospace devices [3]. Consequently, the European Commission (EC) has granted an exemption permitting the continued use of Pb in solders for defense applications [4]. With the increasing miniaturization and integration of devices, solder joints have shrunk in size and increased in density, resulting in higher current density and operating temperatures. Owing to the mismatch of the coefficient of thermal expansion (CTE) between the chip, solder, and substrate, solder joints experience greater thermal stress [5]. SnPb solder, with its low melting point (Tm), is susceptible to creep deformation at room temperature (T/Tm = 0.65), potentially leading to the failure of solder joints [6].

Adding alloy elements to solders can regulate their microstructure and improve their mechanical properties [7]. The addition of elements such as Ag, Cu, In, Sb, and Te has been shown to enhance the creep resistance of pure Pb [8,9]. Specifically, the addition of Sb to Pb results in alloy hardening through continuous precipitation, making it more resistant to impact and wear [8,9]. Sb addition can also improve the creep resistance of pure Sn [10]. Rodney, for instance, incorporated 0 to 8 wt.% Sb into Sn, generating whisker-like SbSn precipitates and thereby reducing the creep rate of a Sn solder at 150 °C [11]. Furthermore, Sb could refine the microstructure of Sn-based solders [12,13]. Adding Sb to a SnBi solder improved the shear strength and creep resistance of solder joints [14,15,16]. Adding Sb nanoparticles to a Sn-9Zn solder increased its tensile strength, with the precipitated Sb3Zn4 acting as a second-phase strengthening agent [17]. Adding Sb to SnAg suppressed the coarsening of Ag_3_Sn and improved the reliability of solder joints [18,19,20]. Furthermore, Sb addition improved the fatigue life of SnAg and SnPb solder joints [21,22].

However, the effects of various Sb contents on eutectic SnPb solder need further investigation. Therefore, the aims of this work were (1) to study the effect of Sb content on the microstructure, tensile, and creep properties of eutectic SnPb solder and (2) to elucidate the creep mechanism by analyzing the microstructure of the solder after creep.

## 2. Materials and Methods

### 2.1. Solder Preparation

Sn, Pb, and Sb (Jinou, Hebei, China) at 99.99% purity were used to prepare the solder ingots. The total masses of the solder were 300.0 g. Alloy elements were weighed according to the proportions outlined in Table 1. They were covered with molten salt to reduce surface oxidation, a combination of KCl and LiCl (Accela, Shanghai, China) in a mass ratio of 1.3:1, and a total mass of 97.0 g. Solder ingots were melted in a resistance furnace at 600 °C for 2 h in a corundum crucible. Molten solders were stirred with a glass rod every 15 min and then cooled in air.

### 2.2. Microstructure Observation

The phase composition of the prepared solders was analyzed using an X-ray diffractometer (XRD, Smartlab, Heddesheim, Germany) at a rate of 5°/min. Scanning electron microscopy and energy-dispersive spectroscopy (SEM and EDS, Hitachi S-4800, Tokyo, Japan) were used to observe the microstructure of the solders at 15 kV. The grain size and grain orientation were examined through electron backscatter diffraction (EBSD, JSM-F100, Tokyo, Japan) at 20 kV with a step size of 0.2 μm. Transmission electron microscopy (TEM, Technai F20, Lonate Pozzolo, Italy) was used to observe the fracture in the solder specimen after creep.

### 2.3. Thermal and Mechanical Properties

**Figure 1:**
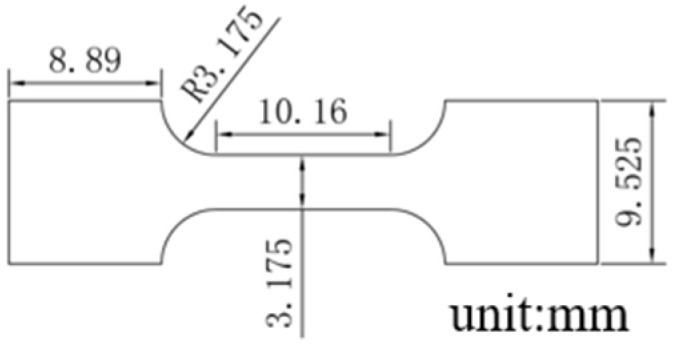
Schematic illustration of tensile test specimens, with a thickness of 1.0 mm.

Thermal properties were assessed using differential scanning calorimetry (DSC, TA DSC25, TA instruments, New Castle, USA) with heating and cooling rates of 10 °C/min. Specimens for tensile tests were laser cut into dog bone shapes from the ingot. The geometry is shown in Figure 1. Then, solders were polished with 400-grit to 2000-grit sandpaper. The tensile properties of the solder were assessed with an Instron-5966 tensile machine (Norwood, MA, USA) at a strain rate of 0.001 s^−1^ at 25 °C and 100 °C. The tensile creep properties were evaluated under constant stress levels of 7, 10, and 15 MPa at 100 °C. Nanoindentation (Keysight Technologie G200, Colorado Springs, CO, USA) was employed to assess the compression creep properties of the solders at 25 °C, using the Constant Strain Rate (CSR) method with a strain rate of 0.02 to 0.05 s^−1^ and a depth of 2000 nm.

## 3. Results

### 3.1. Characterization and Melting Properties of the As-Cast Solder

**Figure 2:**
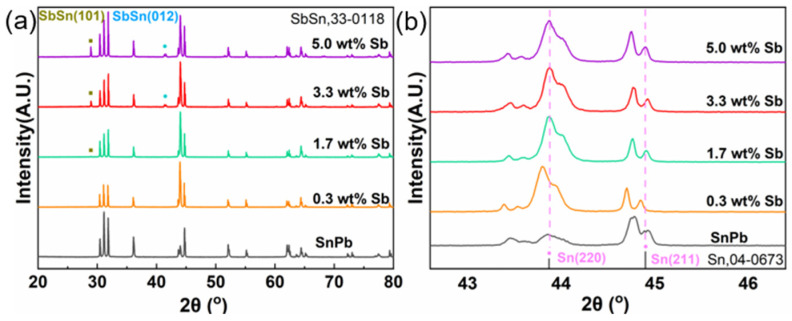
(**a**) XRD results of SnPb solder with 0 to 5.0 wt.% Sb. (**b**) enlarged view of (**a**).

Figure 2 presents the XRD results of SnPb solders with different Sb contents. No peak of SbSn intermetallic compound (IMC) appeared in solder with 0.3 wt.% Sb. When Sb addition exceeded 1.7 wt.%, an SbSn IMC peak emerged, and the peak intensity increased with Sb content. The peritectic reaction of Sn and Sb led to the formation of SbSn IMCs. As shown in the enlarged view, the Sn(220) and Sn(211) peak of the solder with 0.3 wt.% Sb moved leftwards, indicating that the Sn(Sb) solid solution enlarged the Sn lattice. However, the peak of solders with 1.7 to 5.0 wt.% Sb did not show obvious deviation from the original location; this may be attributed to the precipitation of SbSn IMCs, which releases Sb from solid solution.

**Figure 3:**
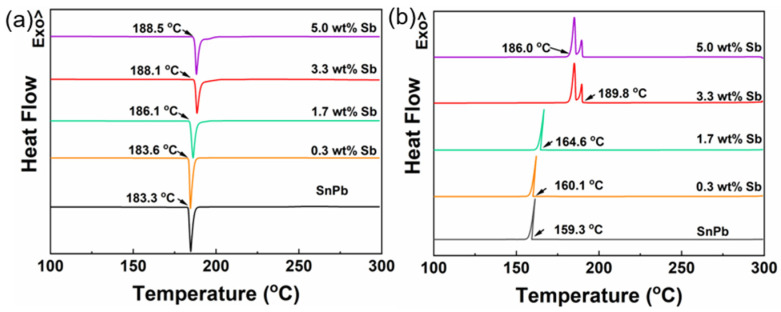
DSC curve of SnPb solder with 0 to 5.0 wt.% Sb; (**a**) heating process; (**b**) cooling process.

Figure 3 illustrates the impact of Sb content on the thermal properties of the SnPb solder. As the Sb content increased, the melting point (Tm), crystallization point (Tx), and cooling range (ΔTs) of the solder increased, as shown in Table 2. SnPb solders with 0.3 to 1.7 wt.% Sb exhibited singular endothermic and exothermic peaks. The increased melting point may be due to Sb having a higher melting point (630 °C) than Sn (232 °C) and Pb (327 °C). In the case of solders containing 3.3 wt.% to 5.0 wt.% Sb, a singular endothermic peak was observed during heating, while two exothermic peaks emerged during cooling. The increased melting point and cooling range may be due to the deviation from SnPb’s eutectic composition and the formation of SbSn IMCs.

**Figure 4:**
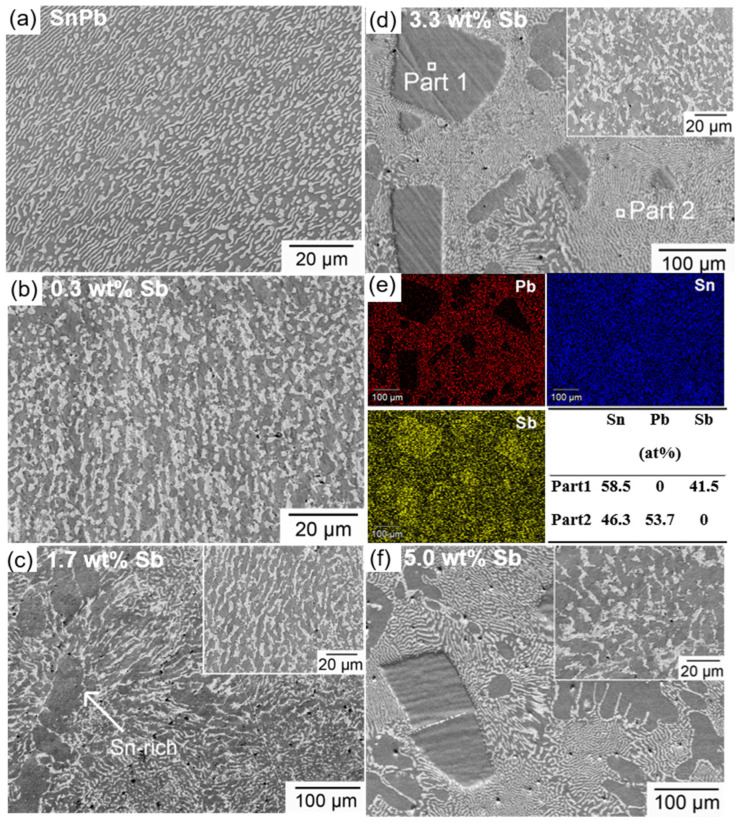
Microstructure of the (**a**) SEM of SnPb solder, (**b**) solder with 0.3 wt.% Sb, (**c**) solder with 1.7 wt.% Sb, (**d**) eutectic SnPb phase of solder with 3.3 wt.% Sb, Part 1 resembles a SbSn IMC, and Part 2 resembles the SnPb eutectic phase, (**e**) EDS results of solder with 3.3 wt.% Sb, (**f**) solder with 5.0 wt.% Sb.

Figure 4 exhibits the microstructure of SnPb solders with various Sb contents. In the solder containing 0.3 wt.% Sb, no SbSn IMCs occurred [23]. The lamellar spacing of the SnPb eutectic phase increased with increasing Sb content. As the Sb content exceeded 1.7 wt.%, a Sn-rich phase appeared. Some Sn-rich phases showed a dendritic morphology due to constitutional supercooling [24]. Notably, when Sb exceeded 3.3 wt.%, bulk SbSn IMCs were created, characterized by a straight and smooth interface with the solder matrix. Part 1 in Figure 4e resembles a SbSn IMC with a Sb-to-Sn atomic ratio of approximately 3:2. Part 2 resembles a SnPb eutectic phase. The higher Pb percentage may be attributed to the expulsion of Pb atoms outward during cooling due to the formation of SbSn IMC, leading to the enrichment of Pb in the eutectic phase.

**Figure 5:**
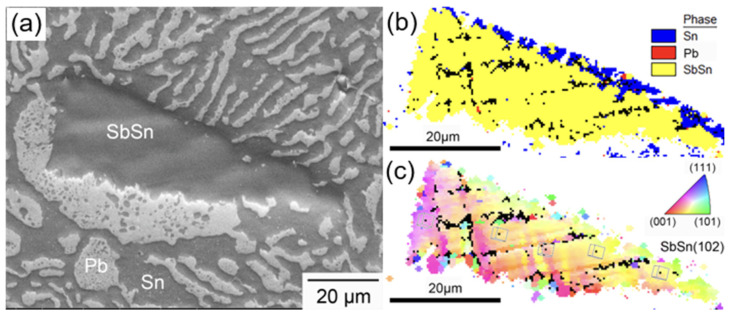
EBSD results of an SbSn IMC. (**a**) SEM; (**b**) phase diagram; (**c**) inverse pole figure of SbSn with similar grain orientations.

As shown in Figure 5, EBSD analysis was carried out to define the type of SbSn phase, which correlated with the SbSn IMC. Most grains in the triangular-shaped SbSn IMC exhibit a similar grain orientation. The (102) crystal planes were approximately parallel to ND, a characteristic that was consistent with the XRD results. The slight difference in grain orientation can be attributed to the rough surface.

### 3.2. Tensile Properties of Solder

**Figure 6:**
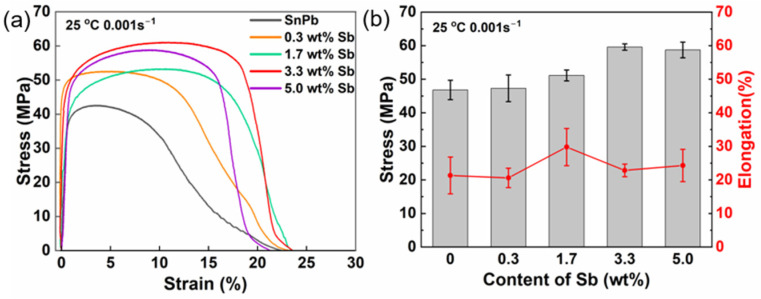
(**a**) Stress–strain curve of SnPb solder with 0 to 5.0 wt.% Sb at 25 °C at a strain rate of 0.01 s^−1^; (**b**) effects of Sb content on ultimate tensile strength and elongation.

Figure 6 shows the stress–strain curve determined in the tensile test conducted at 25 °C at a strain rate of 0.01 s^−1^. Sb addition increased the ultimate tensile strength of the solder and maintained its plasticity. Notably, the tensile strength reached a peak value of 59.6 ± 0.95 MPa for the solder with 3.3 wt.% Sb, a 27.4% increase compared to the eutectic SnPb solder.

The increase in tensile strength can be attributed to both solution-strengthening and composite-strengthening mechanisms. The formation of a Sn(Sb) solid solution enlarges the lattice constant of Sn, inducing lattice disorder and creating an elastic stress field that impedes dislocation movement [25]. The SbSn IMCs were approximately 100 μm in size and distributed unevenly in the solder, in line with the composite-strengthening theory. As SbSn IMCs were harder than the eutectic SnPb phase, they acted as a reinforcement phase and bore more stress, while the softer SnPb phase bore more strain [26]. Subsequently, the solder’s tensile strength improved while the good ductility of the SnPb matrix was retained. The increase in the elongation of the solder with 1.7 wt.% Sb may be due to the transformation from Sn(Sb) solid solution to SbSn IMCs, as solid solution strengthening hinders plastic deformation.

### 3.3. Creep Properties of Solder

**Figure 7:**
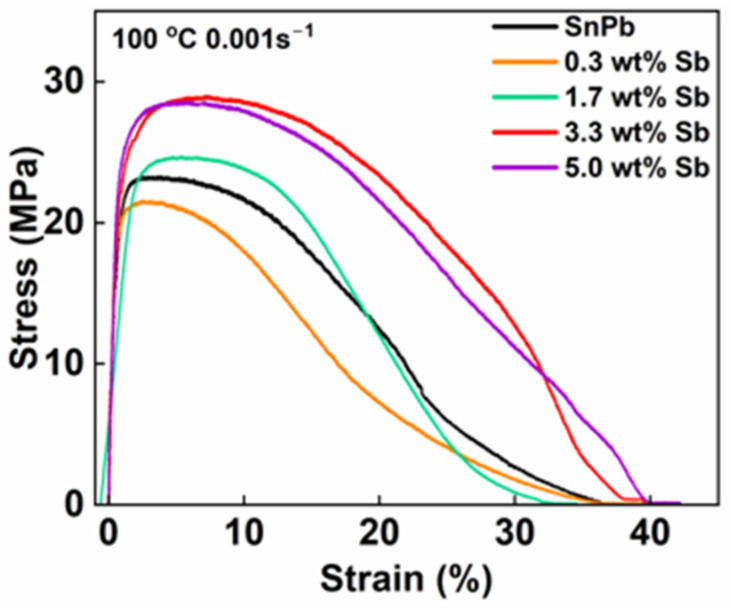
Stress–strain curve of SnPb solders with 0 to 5.0 wt.% Sb at 100 °C at a 0.01 s^−1^ strain rate.

Figure 7 shows the stress–strain curve of the SnPb solder with 0 to 5.0 wt.% Sb stretched at 100 °C at a 0.01 s^−1^ strain rate. The ultimate tensile strength of the SnPb solders was 23.2 MPa with a yield strength of approximately 18 MPa. The tensile strength decreased by 50% and the elongation increased by 60% at 100 °C compared to the tensile test results at 25 °C. This phenomenon aligns with the general trend wherein metals soften at high temperatures [27]. The increase in elongation may be attributed to the increased dislocation slip distance. Importantly, the addition of Sb maintained its strengthening effect on SnPb solders, even at elevated temperatures.

**Figure 8:**
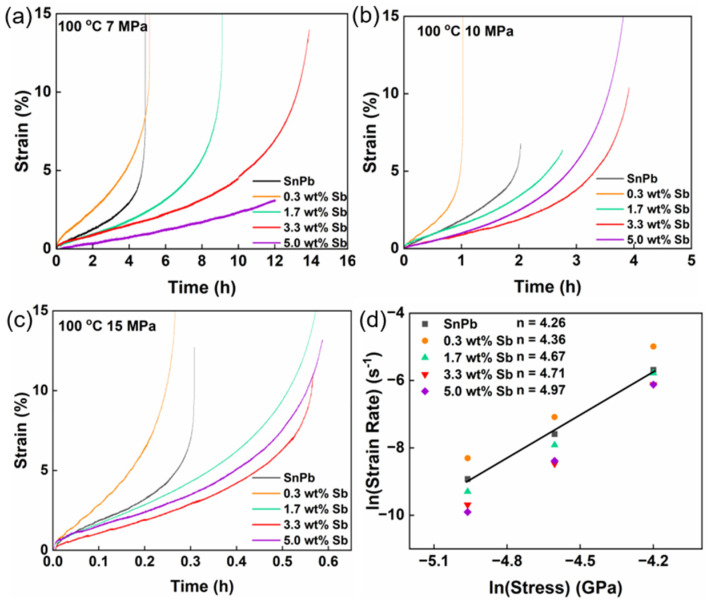
(**a**) Time–strain curve of SnPb solders with 0 to 5.0 wt.% Sb at 100 °C under 7 MPa, (**b**) 10 MPa, and (**c**) 15 MPa; (**d**) ln(strain rate)–ln(stress) curve of solders with 0 to 5.0 wt.% Sb, the stress exponent *n* of SnPb solder is 4.26.

Figure 8a–c show stress–strain curves for the tensile creep of SnPb solders with 0 to 5.0 wt.% Sb at 100 °C under stresses of 7 to 15 MPa. As creep is the plastic deformation below the elastic limit, the maximum applied stress was set below the yield strength of the solder. All solders exhibited steady-state creep and accelerated creep stages. The addition of 1.7 to 5.0 wt.% Sb reduced the steady-state creep rate of SnPb solders and enhanced their creep resistance. Moreover, solders with higher Sb content exhibited lower creep rates, with the impact of Sb addition being more obvious at lower tensile stresses. Furthermore, the Sb content correlated with elongation and the time to fracture. Notably, the solder containing 0.3 wt.% Sb showed a slightly increased creep rate, suggesting that trace amounts of Sb have a limited strengthening effect on Sn, owing to their similar lattice constants, and a minor impact on the solute atmosphere [19]. Additionally, Sb contributed to an increase in the stacking fault energy of Pb, thereby facilitating dislocation glide and recovery in Pb [8,28].

During creep, the processes of work hardening and recovery occur simultaneously. When these rates reach a balance, a steady-state creep stage is established [23]. Various creep models have been developed to describe the stress–strain rate relationship during steady-state creep [24]. A classic, straightforward model is the power law creep model, as represented by Formula (1) [29].

(1) $$\sigma =Aexp(\frac{-\Delta H}{kT})\varepsilon^{n}$$

Here, *ε* is the creep rate, *σ* is the tensile stress, *A* is the material constant, Δ*H* is the activation energy, *k* is Boltzmann’s constant, and *n* is the stress exponent.

Figure 8d illustrates the ln(stress)–ln(strain rate) curve for solders with different Sb contents, where the slope represents the stress exponent *n*. In the case of eutectic SnPb solder, *n* = 4.26, and it increases with increasing Sb content. For eutectic SnPb solder, *n* < 3 is often a sign of superplasticity, observed at high temperatures and under low-stress conditions. Conversely, *n* > 3 suggests a dislocation creep mechanism, typical of higher-stress conditions [30,31,32].

The ability of the Sb addition to enhance the creep resistance of solders can be explained by the composite material-strengthening theory, wherein the soft and hard phases deform unevenly. Hard particles bear more stress, and soft phases undergo greater deformation [33]. Within SnPb solders with 0 to 5.0 wt.% Sb, the eutectic SnPb phase was identified as the softest, followed by the Sn-rich phase, while the SbSn IMCs were characterized as the hardest. The Sb addition decreased the proportion of the eutectic phase, primarily due to the appearance of the Sn-rich phase and SbSn IMCs, making the solder more resistant to creep.

**Figure 9:**
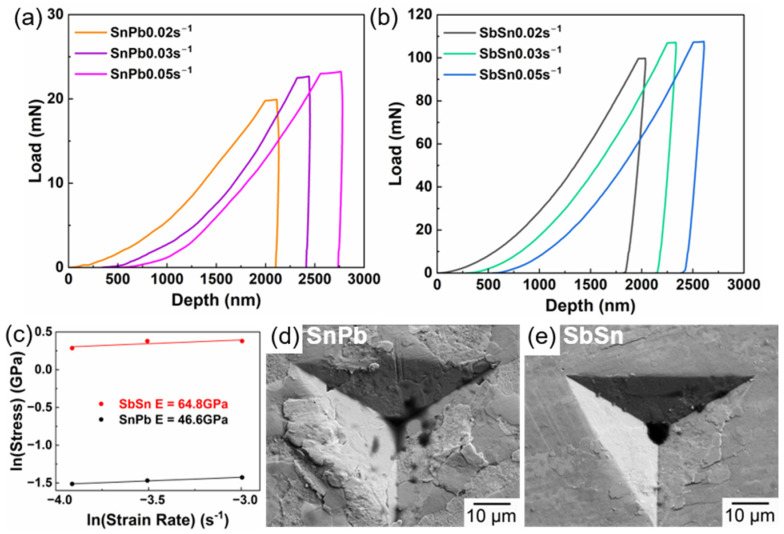
(**a**) Load–time curve of eutectic SnPb phase; (**b**) load–time curve of the SbSn IMC; (**c**) ln(strain rate)–ln(stress) curve; (**d**) morphology of the SnPb phase after nanoindentation at a strain rate of 0.03 s^−1^; (**e**) morphology of the SbSn IMCs after nanoindentation at a strain rate of 0.03 s^−1^.

SbSn IMCs may contribute to the tensile strength and creep resistance through composite strengthening. In Figure 9, the nanoindentation results of the eutectic SnPb phase and SbSn IMCs are provided, with time–load curves adjusted for clarity. Increasing the strain rate leads to increased hardness as a result of work hardening [34]. The hardness of SbSn IMCs was greater than that of the eutectic SnPb phase. The strain rate sensitivity *m* was calculated using Formulas (2) and (3), where *σ* is stress, *ε* is strain, *H* is the hardness, *P* is the maximum load, and *S* is the indentation area. In the case of the SnPb solder, the tensile creep test results are comparable with those of the indentation creep test [35,36,37,38]. SbSn IMCs had a lower strain rate sensitivity (*m* = 0.094) than the eutectic SnPb phase (*m* = 0.097), suggesting that SbSn IMCs contributed to an increased stress exponent *n*. These values of *m* are typical for metal deformation at room temperature [39].

(2) $$\sigma =H=\frac{P}{S}$$

(3) $$m=\frac{ln\sigma }{ln\varepsilon }=\frac{1}{n}$$

Figure 9d,e show the morphology of the eutectic SnPb phase and SbSn IMCs after nanoindentation at a strain rate of 0.03 s^−1^. Extrusion in SnPb was greater than in the SbSn IMC, with multiple cracks along the phase boundaries. This may be due to the SbSn IMCs being a single phase, characterized by fewer phase boundaries than the eutectic phase. Consequently, the reduction in grain boundaries contributed to the limitation of grain boundary glide [40].

**Figure 10:**
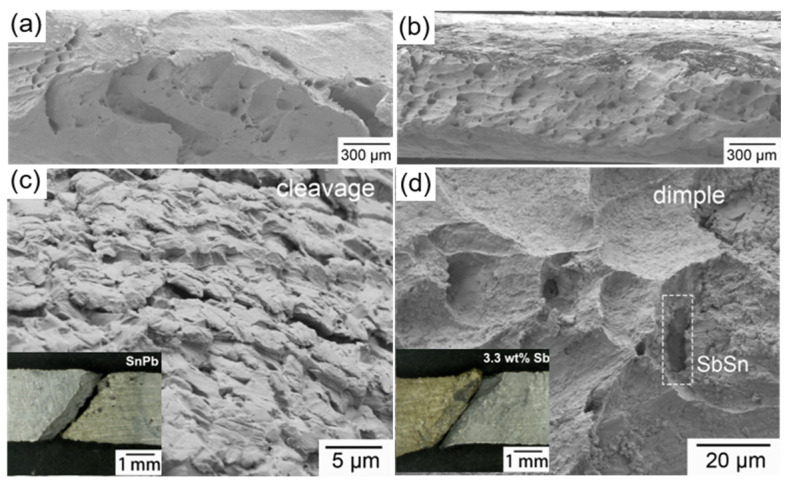
(**a**) SEM of fracture of the SnPb solder; (**b**) fracture of the solder with 3.3 wt.% Sb; (**c**) cleavage steps in SnPb solder fracture; (**d**) dimples in the solder with 3.3 wt.% Sb.

Figure 10 shows the fracture surface of the SnPb solder and the solder with 3.3 wt.% Sb after creep at 100 °C under 15 MPa. Both fracture surfaces formed a 45° angle with the tensile axis, indicative of a fracture triggered by maximum shear stress, a characteristic of plastic fracture [31]. Additionally, necking in the Sb-added solder was more obvious than in the SnPb solder. The fracture morphology of the SnPb solder exhibited slip planes and dimples, while the solder with 3.3 wt.% Sb was mainly composed of dimples. Cleavage steps were observed in the slip planes of the SnPb fracture surface, suggesting an intergranular fracture, while dimples were evident at the fracture of the solder with 3.3 wt.% Sb, which is indicative of a ductile fracture. A SbSn IMC was found at the bottom of a dimple, indicating the hindrance of solder deformation.

From a microscopic perspective, creep deformation under high stress is usually attributed to dislocation movement, where stress-induced work hardening and thermally activated recovery proceed concurrently, resulting in continuous dislocation generation, slip, and offset [41].

**Figure 11:**
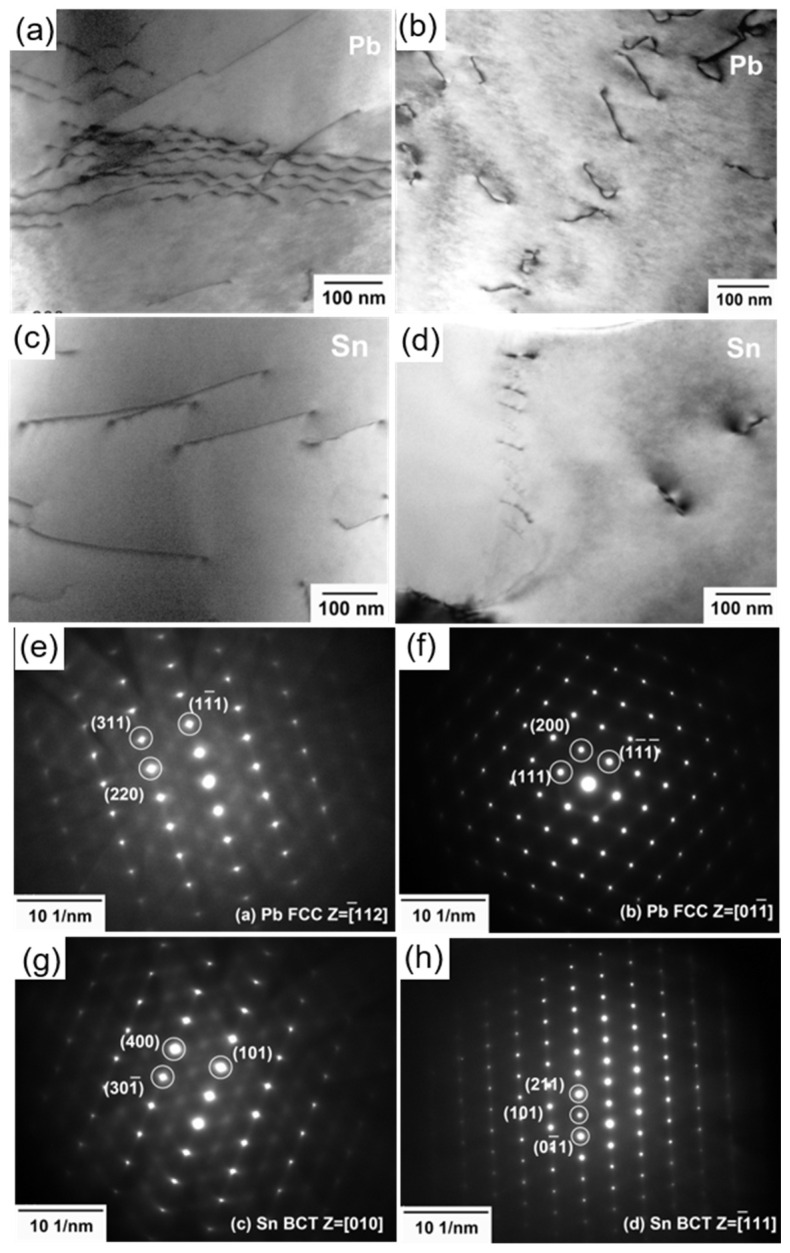
TEM of fracture of the solder with 3.3 wt.% Sb after creep: (**a**,**b**) dislocations in the Pb grains; (**c**,**d**) dislocations in the Sn grains; (**e**) SAED of (**a**); (**f**) SAED of (**b**); (**g**) SAED of (**c**); (**h**) SAED of (**d**).

Figure 11 shows the TEM results of the fracture of the solder with 3.3 wt.% Sb after creep. As shown in Figure 11a, the dislocations in the Pb grain were long and wavy, sharing the same Burgers vector. Dislocations crossing in different directions hindered dislocation slip. A dislocation kink appeared, which is a typical phenomenon at elevated temperatures. During creep, dislocations can glide to release strain [42]. Other dislocations were trapped at the crossing and piled up. However, dislocation crossing in the Sn grain exhibited no signs of stress concentration, with wide spacing and a straight shape. Figure 11d shows a line of dislocation pairs near the phase boundary of the Sn grain. The Pb grain displays a higher dislocation density than the Sn grain, partly because Pb is softer than Sn and bears more deformation. Additionally, Pb’s FCC structure occupies abundant slip systems that facilitate dislocation movement [2,43].

**Figure 12:**
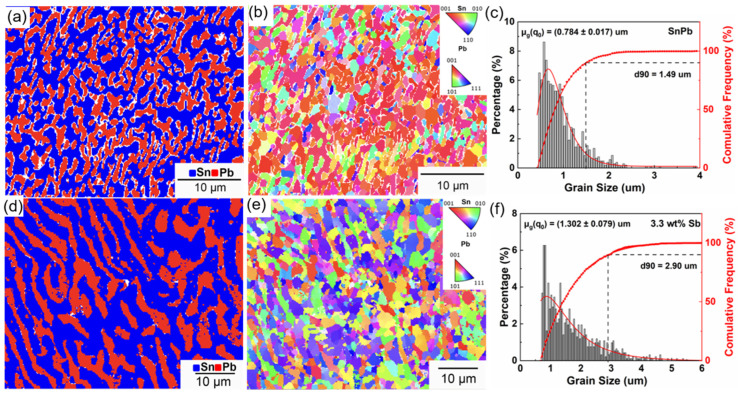
(**a**) Phase map and (**b**) inverse pole figure of the SnPb solder after creep; (**c**) phase map and (**d**) inverse pole figure of the eutectic phase in the solder with 3.3 wt.% Sb after creep; (**e**) grain size distribution of the SnPb solder after creep; (**f**) grain size distribution of the solder with 3.3 wt.% Sb after creep.

Figure 12 shows the EBSD results of the SnPb solder and the solder with 3.3 wt.% Sb after creep under 15 MPa. The average grain sizes of the SnPb solder and the solder with 3.3 wt.% Sb were 0.78 μm and 1.3 μm, while the Sn-rich phase reached approximately 20 μm. The addition of Sb reduced the supercooling of solder and increased the lamellar spacing of the eutectic phase. Typically, grain refinement has a strengthening effect on metals at room temperature since it hinders dislocation motion. However, this effect diminishes at higher temperatures, due to the enhanced diffusion and sliding of atoms along grain boundaries. As grain boundary glide contributes to about 10% of total creep deformation, the Sb addition effectively reduces the grain boundary length, thereby inhibiting creep [44,45,46]. Although the solder with 3.3 wt.% Sb had a longer creep process than the SnPb solder, the difference in the phase-coarsening time was believed to be minimal when aged below 100 °C over a short period [47].

The inverse pole figure of SnPb solder revealed more red grains, indicating the preferred orientations of Sn and Pb. However, the eutectic phase of the solder with 3.3 wt.% Sb showed no evidence of preferred orientation in the inverse pole figure. This may be because there was less grain rotation in the direction of tensile stress, indicating that the Sb solution increased the difficulty of grain boundary sliding [48,49].

**Figure 13:**
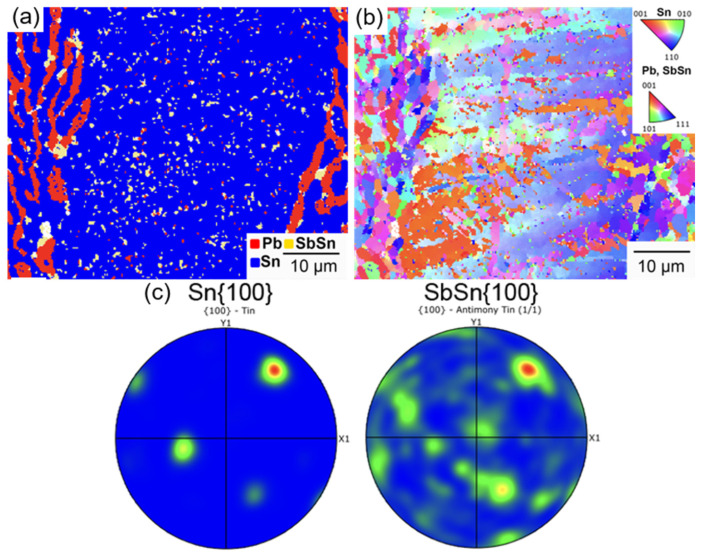
(**a**) Phase map of the Sn-rich phase in the solder with 3.3 wt.% Sb after creep; (**b**) inverse pole figure of the Sn-rich phase; (**c**) pole figure of Sn {100} and SbSn {100}.

As shown in Figure 13, smaller SbSn precipitates were found in the Sn-rich phase and along the phase boundary of solder with 3.3 wt.% Sb. The projection of SbSn {100} and Sn {100} (red spot) overlapped, suggesting an orientation relationship of SbSn {100}//Sn {100}. SbSn particles were prone to grow along the c-direction in a β-Sn matrix [14]. These submicron SbSn precipitates could strengthen the Sn matrix through Orowan stress, thereby contributing to improved creep resistance. According to the Orowan stress theory, when dislocations bypass or cut through precipitates, friction stress is imposed on them, hindering their further movement [27]. The Sn-rich phase primarily consisted of large grains with similar orientations, except at the interface with the eutectic SnPb phase, where fine grains were observed. The observed orientation difference suggests the recrystallization of large Sn grains due to stress concentrating at the interface [22,50].

## 4. Conclusions

The addition of Sb to eutectic SnPb solder induced microstructural changes, ultimately enhancing its mechanical properties.

Solders with added Sb deviated from the eutectic composition. The melting point, crystallization point, and cooling range increased with increasing Sb content. The tensile strength increased with increasing Sb content. This may be due to the precipitation strengthening of SbSn IMCs when Sb content exceeds 1.7 wt.%.

SnPb solders with 0 to 5.0 wt.% Sb exhibited steady-state and accelerated creep stages at 100 °C under stress ranging from 7 to 15 MPa. The strain–stress curve of SnPbSb solders accorded with the power law creep model. When the Sb content exceeded 1.7 wt.%, the creep rate of the solders decreased, and the stress exponent *n* increased from 4.26 to 4.97. The value of the stress exponent suggests a dislocation creep mechanism, while dislocations were observed in Sn and Pb grains after creep. Pb exhibited a higher dislocation density than Sn grains due to its lower hardness. Sb addition resulted in the formation of a Sn(Sb) solid solution and the precipitation of submicron SbSn IMCs from the Sn-rich phase after creep, hindering dislocation slip. SbSn IMCs served as a reinforcing phase due to their hardness compared to the SnPb eutectic phase, strengthening the solder without compromising the ductility of the SnPb matrix. Moreover, the emergence of the Sn-rich phase led to a reduction in the proportion of the eutectic SnPb phase. This decrease in the overall grain boundary length contributed to a reduction in grain boundary sliding, thereby inhibiting creep deformation.

## Figures and Tables

**Table 1:** Composition of SnPb solder with 0 to 5.0 wt.% Sb.

	Sn (wt.%)	Pb (wt.%)	Sb (wt.%)
SnPb	63.0	37.0	0
0.3 wt.% Sb	62.8	36.9	0.3
1.7 wt.% Sb	61.9	36.4	1.7
3.3 wt.% Sb	60.9	35.8	3.3
5.0 wt.% Sb	59.9	35.1	5.0

**Table 2:** Melting point, crystallization point, and cooling range of solders with 0 to 5.0 wt.% Sb.

	Tm (°C)	Tx (°C)	ΔTs (°C)
SnPb	183.3	159.3	3.2
0.3 wt.% Sb	183.6	160.1	3.2
1.7 wt.% Sb	186.1	164.6	4.6
3.3 wt.% Sb	188.1	189.8	9.4
5.0 wt.% Sb	188.5	189.8	9.4

## Data Availability

The raw data supporting the conclusions of this article will be made available by the authors on request.
